# Incidence and Outcomes of Surgically Managed Ectopic Pregnancy in Women With Disabilities: A Population‐Based Cross‐Sectional Study

**DOI:** 10.1111/ppe.70089

**Published:** 2025-11-26

**Authors:** Natalie V. Scime, Beili Huang, Hilary K. Brown, Erin A. Brennand

**Affiliations:** ^1^ Department of Obstetrics and Gynecology, Cumming School of Medicine University of Calgary Calgary Alberta Canada; ^2^ O'Brien Institute for Public Health University of Calgary Calgary Alberta Canada; ^3^ Department of Health and Society University of Toronto Scarborough Toronto Ontario Canada; ^4^ Dalla Lana School of Public Health University of Toronto Toronto Ontario Canada; ^5^ Department of Community Health Sciences, Cumming School of Medicine University of Calgary Calgary Alberta Canada

**Keywords:** disability, ectopic pregnancy, healthcare disparities, National Inpatient Sample

## Abstract

**Background:**

Disparities in the incidence, management, and outcomes of ectopic pregnancy have been documented among marginalised patients; however, there are few data on ectopic pregnancy in women with disabilities.

**Objective:**

To compare the incidence and outcomes of surgically managed ectopic pregnancy in women with and without disability.

**Methods:**

We conducted a population‐based cross‐sectional study using the National Inpatient Sample of discharges from US community hospitals (January 2016–December 2021). We analysed 9769 hospitalisations for surgically managed ectopic pregnancy among females aged 15–44 years. Disability was measured using a published administrative data diagnosis code algorithm. Outcomes were the incidence rate of ectopic pregnancy, surgical management approach (route, tubal removal versus sparing), complications (length of stay [LOS] ≥ 3 days, blood transfusion), and use of more extensive procedures than are standard (hysterectomy, oophorectomy, bilateral salpingectomy, tubal ligation). Weighted analyses were used to generate unadjusted incidence rate ratios (IRR) and outcome risk ratios (RR) from modified Poisson regression adjusted for year of surgery, socio‐demographics, smoking, and comorbidities.

**Results:**

The rate of surgically managed ectopic pregnancy was 2.8 per 1000 obstetric deliveries in disabled females and 2.3 per 1000 in non‐disabled females (IRR 1.26, 95% CI 1.08, 1.45). Compared to non‐disabled females, disabled females more often experienced prolonged LOS (adjusted RR 1.34, 95% CI 1.03, 1.74) and use of extensive procedures (adjusted RR 1.49, 95% CI 1.11, 2.00), including hysterectomy (adjusted RR 1.75, 95% CI 0.91, 3.36), oophorectomy (adjusted RR 1.43, 95% CI 0.96, 2.13), and bilateral salpingectomy (adjusted RR 1.30, 95% CI 0.71, 2.37); however, some estimates were imprecise due to low cell counts.

**Conclusions:**

Disabled women faced slightly higher rates of surgically managed ectopic pregnancy and use of more extensive surgical procedures, including sterilisation. Targeted patient education on ectopic pregnancy and equity‐focused guidance for surgeons may be beneficial.

## 1 Introduction

Ectopic pregnancy is the implantation of an embryo outside the uterine cavity, most often in the distal fallopian tube [1], and is a persistent public health problem. Occurring in roughly 2% to 3% of all pregnancies [2, 3, 4, 5], ectopic pregnancy is a gynecologic emergency with substantial imminent risk of maternal morbidity and mortality [6], as well as subsequent risks of adverse fertility and perinatal outcomes [7]. As with all forms of pregnancy loss, ectopic pregnancy can be highly distressing and emotionally complex for women to navigate [8].

Early diagnosis of ectopic pregnancy is vital for prompt management. Clinical assessment relies heavily on presenting symptoms (lower abdominal pain or vaginal bleeding), physical exam, and patient risk factors to determine high suspicion for ectopic pregnancy warranting transvaginal sonogram and quantitative serum human chorionic gonadotropin (hCG) testing. Hospitalisation is required in the majority of cases to surgically manage ectopic pregnancy due to either hemodynamic instability, patient preference, or contraindications to outpatient medical management with methotrexate [4]. Importantly, disparities in the management and complications of ectopic pregnancy have been commonly documented among marginalised patients, such as those with low socioeconomic status or racial diversity [9, 10, 11, 12, 13, 14], highlighting the need for equity, diversity, inclusivity, and accessibility considerations in ectopic pregnancy care.

Approximately 1 in every 5 reproductive‐aged women has a medical condition that gives rise to disability [15, 16], including physical, sensory, and intellectual impairments. Research has shown that women with disabilities have access to a narrow range of contraception options [17], face disproportionately higher rates of pregnancy‐related morbidity [18, 19] and certain gynecologic surgeries such as hysterectomy [20, 21, 22, 23], and regularly confront ableism and marginalisation in their pursuit of reproductive care [24, 25]. There is an important cultural backdrop to these trends. Between 1920 and 1980, women with disabilities were subjected to forced or coerced sterilisation at alarming rates under the guise of legitimate medical care or concern for patient welfare [26]. This practice was largely discontinued in the late 20th century following legal challenges and recognition as a violation of human rights. However, uninformed sterilisation and other reproductive care disparities rooted in eugenics continue to exist for patients with disabilities.

At present, there are few data on the epidemiology of ectopic pregnancy in women with disabilities. The acute nature of the condition combined with entrenched biases about the reproductive capacity of women with disabilities may create a precarious context for disparities in care. The aim of this study was therefore to compare the incidence and outcomes of hospitalisations for surgically managed ectopic pregnancy in women with and without disabilities.

## 2 Methods

### 2.1 Sample

We conducted a cross‐sectional analysis of all hospitalisations for reproductive‐aged females 15 to 44 years old receiving surgical care for an ectopic pregnancy from January 1, 2016, to December 31, 2021, in the National Inpatient Sample (NIS). The NIS is a population‐based database in the Healthcare Cost and Utilisation Project (HCUP), sponsored by the Agency for Healthcare Research and Quality [27]. As the largest all‐payer database of hospitalisations in the United States, the NIS is composed of a 20% sample of discharges from all community hospitals in participating states, excluding rehabilitation and long‐term acute care hospitals. Each NIS record represents a single de‐identified hospitalisation encounter, including patient demographics, hospital characteristics, and diagnosis and procedure codes reported using the *International Classification of Diseases*, *Tenth Revision*, *Clinical Modification* (ICD‐10‐CM). NIS variables and ICD‐10‐CM codes used to define all study variables are outlined in Table S1. Herein, we refer to patients by their biological sex (females), given that data on gender identity were unavailable [28].

### 2.2 Exposure

Disability was measured by applying an administrative data diagnosis code algorithm to each inpatient record for surgically managed ectopic pregnancy to ascertain the presence of a chronic and function‐limiting health condition in the pregnant patient [18]. Females were classified as having one or more physical (i.e., musculoskeletal disorder, neurologic disorder, permanent injury, or congenital anomaly), sensory (i.e., hearing loss or vision loss), and/or intellectual or developmental (i.e., intellectual disability or autism spectrum disorder) disabilities and compared to females with no disability as the reference group.

### 2.3 Outcome

Incidence of ectopic pregnancy was defined as the number of hospitalisations for surgically managed ectopic pregnancy per 1000 hospitalisations for obstetric deliveries [29, 30, 31]. The NIS was used to estimate the denominator of the number of obstetric deliveries among females with and without disability. Outcomes were surgical management including route of surgery (open versus minimally invasive) and nature of surgery (salpingectomy [tubal removal] versus salpingostomy [tubal sparing]); complications including prolonged length of stay (≥ 3 days; LOS) and blood transfusion; and use of more extensive procedures than are standard including hysterectomy, oophorectomy (bilateral or unilateral), bilateral salpingectomy, or tubal ligation.

### 2.4 Covariates

Covariates were predetermined based on dataset availability and relevance to health disparities in females with disabilities and to ectopic pregnancy epidemiology. These included patient age, race and ethnicity, type of health insurance, median household income quartile according to ZIP code, past and current smoking status, selected comorbidities from the Elixhauser Index such as cardiometabolic diseases, psychiatric illnesses, and obesity, and gynecologic diseases (Table S1).

### 2.5 Statistical Analysis

All descriptive and inferential analyses accounted for the NIS sampling design with specialised statistical commands in R version 4.2.2 for complex survey data incorporating sampling weights (to represent the national US population), clustering, and stratification. Sampling weights were derived by the HCUP by dividing the number of universe discharges by the number of sampled discharges within each NIS stratum (defined by hospital census division, rural/urban location, bed size, teaching status, and ownership).

We used frequencies and proportions to describe the baseline characteristics of females hospitalised for surgical management of ectopic pregnancy with and without disabilities and compared them using standardised differences. First, we calculated incidence rates to report the unadjusted incidence rate ratio (IRR) and 95% confidence intervals (CI) for the association between disability status and ectopic pregnancy rate, including overall IRR estimates and age‐stratified IRR estimates. Next, we used modified Poisson regression to report risk ratios (RR) and 95% CI for the associations of disability status with outcomes related to surgical management, complications, and use of extensive procedures. Models were specified using a multi‐step approach. The main model adjusted for year of surgery, socio‐demographic factors (age, race/ethnicity, health insurance, median household income quartile), and smoking; one supplementary model additionally adjusted the main model for comorbidity profiles (cardiometabolic diseases, psychiatric illnesses, obesity and gynecologic diseases); and another supplementary model excluded potential cases of Caesarean scar pregnancy (which may be a driver of ectopic pregnancy complications and use of hysterectomy) from the main model. This approach was used to consider the relative impact of each set of patient factors on the associations under study and to monitor model stability with the addition of variables, given the rare exposure prevalence and modest sample size. To explore the impact of unmeasured confounding, we calculated e‐values for the main model, defined as the minimum strength of association that an unmeasured confounder would need to have with both the exposure and outcome to fully explain away the observed exposure‐outcome association [32].

### 2.6 Missing Data

Missing covariate data were minimal (3.7%; Table 1) and handled through the use of a separate variable category.

**TABLE 1:** Characteristics of National Inpatient Sample records for patients with and without disabilities who received surgical treatment for an ectopic pregnancy between 2016 and 2021.

Characteristic	No disability No. (%)	Disability No. (%)	ASD
Total	*N* = 9591	*N* = 178 (1.8)	—
Age (years)			
15–19	298 (3.1)	NR (1.0–2.0)	0.14
20–24	1425 (14.9)	26 (14.6)	0.01
25–29	2677 (27.9)	40 (22.5)	0.13
30–34	2864 (29.9)	55 (30.9)	0.02
35–39	1857 (19.4)	42 (23.6)	0.10
40–44	470 (4.9)	13 (7.3)	0.10
Race/ethnicity			
Asian/other	888 (9.3)	NR (3.0–4.0)	0.22
Black	2800 (29.2)	51 (28.7)	0.01
Hispanic	2562 (26.7)	30 (16.9)	0.24
White	3117 (32.5)	87 (48.9)	0.34
Missing	224 (2.3)	NR (1.0–2.0)	0.05
Insurance payer			
Medicaid or medicare	4393 (45.8)	92 (51.7)	0.12
Private insurance	3721 (38.8)	65 (36.5)	0.05
Other	1462 (15.2)	20 (11.2)	0.12
Missing	15 (0.2)	NR (0.0–1.0)	0.07
Median household income for ZIP code			
Quartile 1 (low)	3602 (37.6)	64 (36.0)	0.03
Quartile 2	2318 (24.2)	33 (18.5)	0.14
Quartile 3	2093 (21.8)	51 (28.7)	0.16
Quartile 4 (high)	1462 (15.2)	24 (13.5)	0.05
Missing	116 (1.2)	NR (3.0–4.0)	0.14
Comorbidities			
Diabetes	158 (1.6)	NR (2.0–3.0)	0.08
Hypertension	331 (3.5)	15 (8.4)	0.21
Cardiovascular disease	368 (3.8)	NR (3.0–4.0)	0.00
Depression	257 (2.7)	29 (16.3)	0.48
Anxiety	393 (4.1)	30 (16.9)	0.43
Substance use disorder (alcohol or drugs)	340 (3.5)	11 (6.2)	0.12
Obesity	748 (7.8)	25 (14.0)	0.20
Endometriosis	214 (2.2)	NR (4.0–5.0)	0.13
Uterine fibroids	299 (3.1)	NR (3.0–4.0)	0.01
Abnormal uterine bleeding	152 (1.6)	NR (2.0–3.0)	0.05
Pelvic inflammatory disease	446 (4.7)	14 (7.9)	0.13
Adnexal pathology	1095 (11.4)	22 (12.4)	0.03
Smoking status			
Never	7819 (81.5)	114 (64.0)	0.40
Former smoker	515 (5.4)	25 (14.0)	0.30
Current smoker	1257 (13.1)	39 (21.9)	0.23

*Note:* > 0.10 indicates an important difference between women with versus without disability.

Abbreviations: ASD, absolute standardised difference; No. (%), unweighted number (weighted proportion); NR, numbers < 11 were not reported.

### 2.7 Ethics Approval

Following the Tri‐Council Policy Statement (Article 2.2), this secondary analysis using publicly available data was exempt from ethical review and approval.

## 3 Results

From the NIS, we analyzed 9769 hospitalisations for female patients aged 15 to 44 years hospitalised for surgical management of ectopic pregnancy and estimated a denominator sample of 4,317,508 obstetric deliveries (Figure S1). Among ectopic pregnancies, there were 178 hospitalisations among females with a disability (weighted prevalence of 1.8%) and 9591 hospitalisations among females without a disability; by type, physical disability was most common (1.7%), followed by sensory (0.2%) or intellectual or developmental (< 0.1%) disabilities, and multiple disabilities (0.1%). Females with a disability were older and more likely to be of White race and ethnicity, to be receiving Medicaid or Medicare insurance, to have certain comorbidities (e.g., hypertension, depression), and to be former or current smokers than females without a disability (Table 1 for the ectopic pregnancy sample, Table S2 for the obstetric deliveries denominator sample).

The overall rate of surgically managed ectopic pregnancy was 2.8 per 1000 obstetric deliveries in females with a disability and 2.3 per 1000 obstetric deliveries in females without a disability. Age stratification showed that ectopic pregnancy rates and IRRs were consistently higher in females with a disability than without a disability in those aged 20 years and older (Table 2).

**TABLE 2:** Incidence of surgically managed ectopic pregnancy by disability status, National Inpatient Sample 2016–2021.

Disability	Unweighted No.	Weighted Incidence rate Per 1000 deliveries	Unadjusted IRR (95% CI)
Overall			
No disability	9591	2.3	1.00 (Reference)
Any disability	178	2.8	1.26 (1.08, 1.45)
*Stratified by age*			
≤ 19 years			
No disability	298	1.5	1.00 (Reference)
Any disability	NR	< 1.0	NR
20–24 years			
No disability	1425	1.7	1.00 (Reference)
Any disability	26	2.4	1.36 (0.83, 1.88)
25–29 years			
No disability	2677	2.2	1.00 (Reference)
Any disability	40	2.4	1.09 (0.75, 1.44)
30–34 years			
No disability	2864	2.3	1.00 (Reference)
Any disability	55	3.0	1.30 (0.96, 1.65)
35–39 years			
No disability	1857	2.9	1.00 (Reference)
Any disability	42	3.7	1.28 (0.89, 1.67)
≥ 40 years			
No disability	470	3.5	1.00 (Reference)
Any disability	13	4.7	1.32 (0.59, 2.05)

*Note:* The denominator for ectopic pregnancy incidence rate is number of obstetric deliveries in National Inpatient Sample over the study period.

Abbreviations: CI, confidence interval; IRR, incidence rate ratio; NR, not reported due to low cell counts.

Overall, females with a disability hospitalised for surgical management of ectopic pregnancy were more likely to have prolonged lengths of stay (≥ 3 days) than females without a disability after adjusting for socio‐demographics and smoking. Females with a disability experienced elevated rates of more extensive procedures than are standard, including hysterectomy, oophorectomy, and bilateral salpingectomy. Point estimates remained elevated after adjusting for socio‐demographics and smoking, with 95% CI ranging from a null to large increase in the rate of individual extensive procedures, and *e*‐values suggested that a moderate‐to‐large magnitude of unmeasured confounding would be required to fully explain these observed differences (Table 3). Use of minimally invasive (versus open) surgery, tubal‐sparing (versus tubal removal) surgery, and blood transfusion were similar among females with and without a disability. Results were largely similar in supplementary models; further adjustment for comorbidities slightly attenuated point estimates, whereas excluding potential cases of Caesarean scar pregnancy did not materially impact point estimates or 95% CI (Table S3).

**TABLE 3:** Outcomes of surgically managed ectopic pregnancy by disability status, National Inpatient Sample 2016–2021.

Disability	No. (%)	Unadjusted RR (95% CI)	Adjusted RR (95% CI)	*E*‐value
Open (vs. minimally invasive) route of surgery				
No disability	3876 (40.4)	1.00 (Reference)	1.00 (Reference)	
Any disability	71 (39.9)	0.99 (0.83, 1.18)	0.98 (0.83, 1.17)	1.14
Tubal removal (vs. tubal sparing) procedure				
No disability	7650 (82.9)	1.00 (Reference)	1.00 (Reference)	
Any disability	131 (79.9)	0.96 (0.89, 1.04)	0.96 (0.89, 1.04)	1.25
Prolonged LOS				
No disability	1715 (17.9)	1.00 (Reference)	1.00 (Reference)	
Any disability	43 (24.2)	1.35 (1.04, 1.76)	1.34 (1.03, 1.74)	2.02
Blood transfusion				
No Disability	1444 (15.1)	1.00 (Reference)	1.00 (Reference)	
Any Disability	25 (14.0)	0.93 (0.65, 1.34)	0.95 (0.66, 1.37)	1.30
Composite extensive procedures^a^				
No disability	1239 (12.9)	1.00 (Reference)	1.00 (Reference)	
Any disability	37 (20.8)	1.61 (1.20, 2.15)	1.49 (1.11, 2.00)	2.34
Hysterectomy				
No disability	248 (2.6)	1.00 (Reference)	1.00 (Reference)	
Any disability	NR (5.0–6.0)	1.96 (1.02, 3.74)	1.75 (0.91, 3.36)	2.89
Oophorectomy				
No disability	803 (8.4)	1.00 (Reference)	1.00 (Reference)	
Any disability	22 (12.4)	1.48 (1.00, 2.19)	1.43 (0.96, 2.13)	2.20
Bilateral salpingectomy				
No disability	331 (3.5)	1.00 (Reference)	1.00 (Reference)	
Any disability	NR (5.0–6.0)	1.63 (0.88, 3.00)	1.30 (0.71, 2.37)	1.92

*Note:* Adjusted models controlled for patient age, race/ethnicity, insurance type, median household income quartile, smoking status, and year of surgery.

Abbreviations: CI, confidence interval; No. (%), unweighted number (weighted proportion); NR, not reported due to low cell counts; RR, risk ratio.

^a^ Use of more extensive procedures than standard, including hysterectomy, oophorectomy (bilateral or unilateral), bilateral salpingectomy, or tubal ligation.

## 4 Comment

### 4.1 Principal Findings

In this population‐based study, the rate of hospitalisation for ectopic pregnancy was slightly higher in females with a disability than without a disability, and specifically in those aged 20 years and older. Females with a disability more often experienced prolonged LOS and higher rates of hysterectomy, oophorectomy, and bilateral salpingectomy than females without a disability despite similar use of surgical routes, the use of salpingectomy versus salpingostomy, and blood transfusion between groups. However, low outcome rates and wide 95% CI for these more extensive procedures limit the certainty of these findings. These data suggest clinically important disparities in the hospitalisation and management of ectopic pregnancy among women with a disability.

### 4.2 Strengths of the Study

Strengths of this study include the large and nationwide sample, multi‐year data and inclusion of all‐payer hospitalisations throughout the United States in a variety of hospital settings, which collectively support the generalisability of our findings. We used a published and expert physician‐reviewed health administrative data algorithm to capture disability, which enabled us to capture a broad scope of disabling health conditions [18].

### 4.3 Limitations of the Data

Our study is not without limitations. Measurement of disability using administrative health data is commonplace and generally valid for epidemiologic research, yet can be subject to several potential mechanisms for misclassification. For non‐differential mechanisms of bias, measurement may have been affected by reduced sensitivity from use of diagnostic codes at a single hospitalisation (vs. multiple encounters over a lookback period) [33, 34] and reduced specificity given the medical model of disability assumption that certain diagnoses universally result in functional limitations. For differential mechanisms of bias, disability may have been more often recorded for patients who experienced poorer health outcomes if viewed as a key contributor, or conversely, less often recorded if a large volume of higher priority disease codes filled the available diagnoses array in the database. The net impact of misclassification bias is thus challenging to qualitatively or quantitatively estimate, but remains an important caveat to our findings. Factors such as patient medical history, gestational age of the pregnancy, site of pregnancy implantation, hemodynamic stability, and post‐operative pain were not available in this data source and thus not included in our analysis yet hold important explanatory potential for future research. Since ectopic pregnancy is rare we often faced small cell counts and imprecise model estimates despite pooling multiple years of data, wherein point estimates represented clinically important outcome differences, but 95% CIs were wide and ranged from a null to large difference in outcome rates. Finally, our analysis of the NIS focused only on inpatient hospitalisations, and thus ectopic pregnancies managed on an outpatient basis were not captured.

### 4.4 Interpretation

Existing studies on ectopic pregnancy care in marginalised patients have focused on socioeconomic status and racial diversity [9, 10, 11, 12, 13, 14]. For example, Hsu et al. (2017) analyzed 62,588 US women with tubal ectopic pregnancy between 2006 and 2015 and found that, among those who underwent surgery, tubal conserving surgery was less likely among Black and Hispanic patients versus White patients and among Medicaid and uninsured patients versus commercially insured patients [9]. Stulberg et al. (2011) analyzed 13,007 ectopic pregnancy hospitalisations in Illinois, United States in 2000–2006 and found that ectopic pregnancy patients with Medicaid, Medicare, or no insurance versus private insurance were more likely to experience surgical sterilisation and prolonged length of stay [13]. Our study expands this literature by adding the construct of disability, which increasingly affects reproductive‐aged women and is consistently associated with disparities in pregnancy care and outcomes.

The elevated ectopic pregnancy rate we observed in women with disabilities may have both biological and social explanations. Biologically, ectopic pregnancy is generally caused by fallopian tube abnormalities that impede embryo passage into the uterine cavity. Women with disabilities are more likely to have acquired risk factors [35, 36, 37] for structural tubal damage, such as a history of sexually transmitted infection [38, 39] and endometriosis [40], and for tubal dysfunction, such as advanced age, smoking, and use of certain medications such as benzodiazepines [15, 41]. Socially, women with disabilities often experience inadequate preconception and prenatal care [42]. These patients may therefore face access barriers to early pregnancy primary care and have less opportunity for education about what early pregnancy symptoms (i.e., lower abdominal pain, vaginal bleeding) should be promptly assessed, resulting in greater hospital visits for advanced ectopic pregnancy [43].

We also observed that prolonged length of stay following surgically managed ectopic pregnancy was more common in females with disabilities, which could be attributed to higher surgical complexity, slower post‐operative recovery due to pain or functional status in these patients, or challenges arranging discharge support to assist with activities of daily living. We also observed greater use of hysterectomy, oophorectomy (> 95% were unilateral), and bilateral salpingectomy in females with disabilities. While the adjusted 95% CI for differences in these procedures by disability status enclosed the null, it is unlikely that all three procedures would be simultaneously elevated due to chance alone as they collectively involve removing reproductive organs. Use of hysterectomy, oophorectomy, and bilateral salpingectomy may be explained by some degree of higher case complexity, noting that point estimates attenuated with additional control for patient comorbidities. Although patient request for voluntary sterilisation due to lack of desire for future fertility is also possible, performing such a procedure during an emergent medical presentation is widely regarded as ethically inappropriate due to concerns about informed consent, decision‐making capacity under distress, and adherence to medical principles of autonomy and non‐maleficence [44, 45]. Given the legacy of eugenics and ableism in reproductive healthcare, it is also possible that our results reflect surgeon or systemic bias, lowering the threshold for these invasive and potentially inappropriate procedures such as sterilisation in the emergent care of ectopic pregnancy in a disabled patient. These data align with prior reports of higher rates of more extensive gynecologic procedures than are standard and surgical sterilisation in women with versus without disabilities [20, 21, 22, 23].

Our findings reinforce the importance of recognising females with a disability as a group experiencing far‐reaching reproductive health disparities. While disability itself is unlikely to be a causal risk factor for ectopic pregnancy, patients with a disability may have a higher prevalence of underlying biological and social risk factors that cumulatively increase their risk of hospitalisation for ectopic pregnancy. Females with a disability may therefore benefit from targeted education on ectopic pregnancy risks and timeliness of healthcare in the event of possible symptoms. Given that roughly 50% of pregnancies in the United States are unintended [46], such education should be a component of periconceptional care and general sexual and reproductive care, such as during contraception counseling and cervical cancer screening, when discussions surrounding heterosexual activity and risk of pregnancy take place. These data also strengthen growing calls for judicious and equitable use of more extensive surgical procedures than are standard, including sterilisation, in females with disabilities. Surgeons should ensure that the decision to use hysterectomy, oophorectomy, or bilateral salpingectomy in the surgical management of ectopic pregnancy is strongly supported by medical necessity and should be vigilant against their own ableist biases when making clinical decisions. This is particularly relevant given increasing evidence on the potential risks of early hysterectomy and oophorectomy for women's long‐term health [47, 48, 49].

## 5 Conclusions

This population‐based study of hospitalisations in the United States showed women with a disability face slightly higher rates of surgically managed ectopic pregnancy and may receive more extensive procedures such as hysterectomy, oophorectomy, and bilateral salpingectomy at a disproportionately higher frequency than women without a disability. Findings support the need for targeted education on ectopic pregnancy risks and symptoms among heterosexually active females with a disability and on equity‐focused approaches to the surgical management of ectopic pregnancy among gynecologic surgeons.

## Author Contributions

N.V.S. and E.A.B. conceptualised the study access to the dataset. N.V.S. and B.H. drafted the analysis plan and conducted the analysis. N.V.S. wrote the initial manuscript draft. H.K.B. and E.A.B. contributed to data interpretation and critically reviewed the manuscript. All authors approved the final version of the manuscript and submission.

## Ethics Statement

In accordance with the Tri‐Council Policy Statement (Article 2.2), this secondary analysis using publicly available data was exempt from ethical review and approval.

## Conflicts of Interest

The authors declare no conflicts of interest.

## Supporting information


**Data S1:** ppe70089‐sup‐0001‐Supinfo1.docx.

## Data Availability

The National Inpatient Sample data is available for purchase through the HCUP Central Distributor (www.hcup‐us.ahrq.gov).
