# Supplementary material for: Incidence and Outcomes of Surgically Managed Ectopic Pregnancy in Women With Disabilities: A Population‐Based Cross‐Sectional Study
Source: Paediatr Perinat Epidemiol. 2025 Nov 26;40(3):378–85. doi: 10.1111/ppe.70089 (PMC13124687; doi:10.1111/ppe.70089)
Supplement: Supplementary file 1 — Data S1: ppe70089‐sup‐0001‐Supinfo1.docx. [file PPE-40-378-s001.docx]

**Supplementary Material**

**Incidence and outcomes of surgically managed ectopic pregnancy in women with disabilities: A population-based cross-sectional study**

eFigure 1. Flow diagram of included hospitalizations from the National Inpatient Sample, 2016-2021

eTable 1. Diagnostic and procedure codes used to define study variables

eTable 2. Characteristics of National Inpatient Sample records for patients with and without disabilities who had an obstetric delivery between 2016-2021

eTable 3. Outcomes of surgically managed ectopic pregnancy by disability status, National Inpatient Sample 2016-2021, further assessed by additionally adjusting for comorbidities and excluding cases of Cesarean scar pregnancy

**eFigure 1. Flow diagram of included hospitalizations from the National Inpatient Sample, 2016-2021 (unweighted frequencies)**


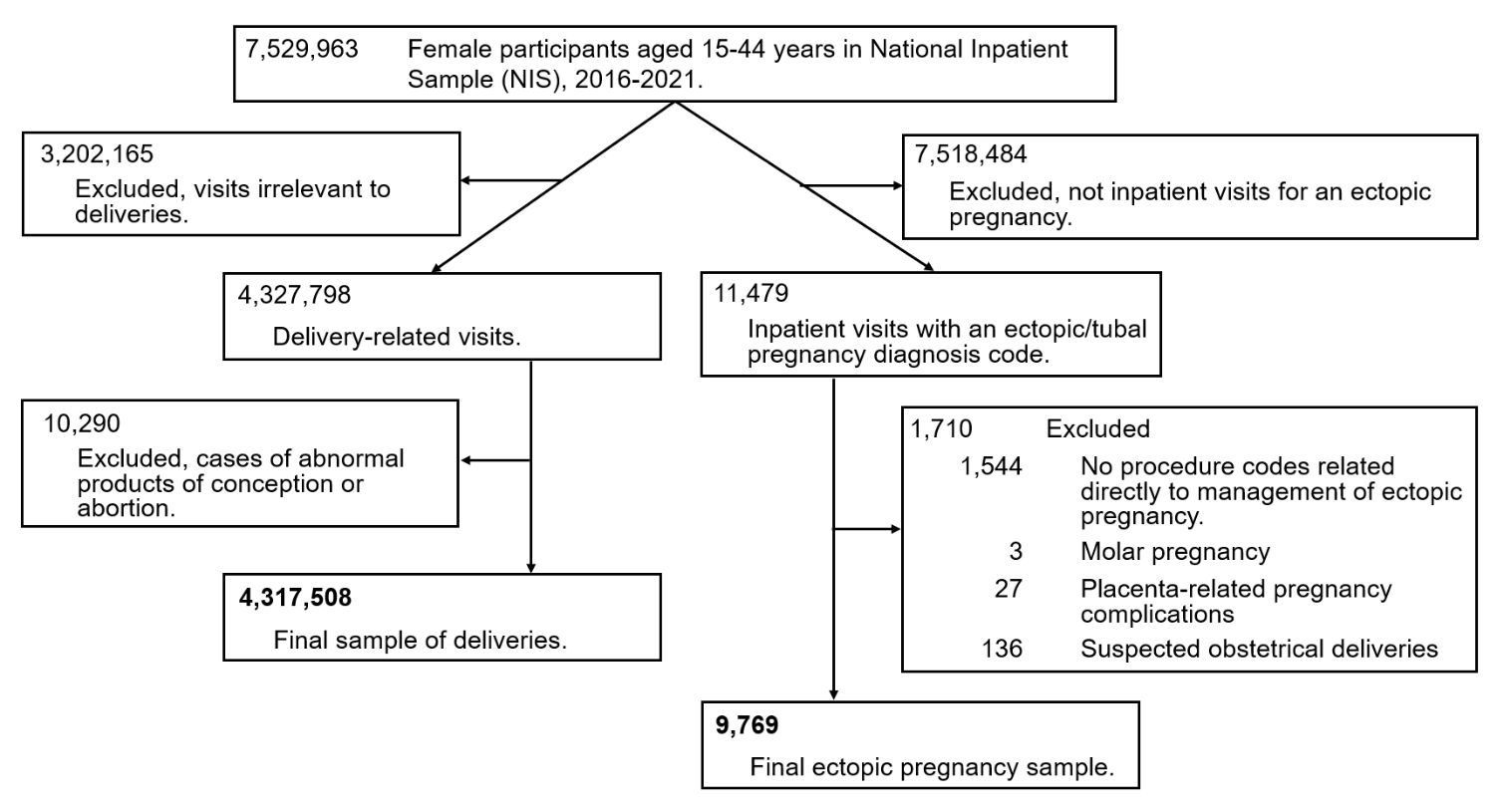


**eTable 1. Diagnostic and procedure codes used to define study variables**

| Study Variable | Diagnostic and/or Procedure Codes |
| --- | --- |
| Sample |  |
| Ectopic pregnancy | ICD-10-CM O00 *AND* at least one PCS code for surgical management of ectopic pregnancy  Prespecified ICD-10-PCS: 0U9700Z, 0U970ZX, 0U970ZZ, 0U973ZZ, 0U9740Z, 0U974ZX, 0U974ZZ, 0U9770Z, 0U977ZX, 0U977ZZ, 0U9780Z, 0U978ZX, 0U978ZZ, 0UC70ZZ, 0UC73ZZ, 0UC74ZZ, 0UC77ZZ, 0UC78ZZ, 10D20ZZ, 10D24ZZ, 10D27ZZ, 10D28ZZ, 10T20ZZ, 10T23ZZ, 10T24ZZ, 10T27ZZ, 10T28ZZ, 0U550ZZ, 0U553ZZ, 0U554ZZ, 0U557ZZ, 0U558ZZ, 0U560ZZ, 0U563ZZ, 0U564ZZ, 0U567ZZ, 0U568ZZ, 0UB50ZZ, 0UB53ZZ, 0UB54ZZ, 0UB57ZZ, 0UB58ZZ, 0UB60ZZ, 0UB63ZZ, 0UB64ZZ, 0UB67ZZ, 0UB68ZZ, 0UT50ZZ, 0UT54ZZ, 0UT57ZZ, 0UT58ZZ, 0UT5FZZ, 0UT60ZZ, 0UT64ZZ, 0UT67ZZ, 0UT68ZZ, 0UT6FZZ, 0U9500Z, 0U950ZX, 0U950ZZ, 0U950ZZ, 0U953ZZ, 0U9540Z, 0U954ZX, 0U954ZZ, 0U9570Z, 0U957ZX, 0U957ZZ, 0U957ZZ, 0U9580Z, 0U958ZX, 0U958ZZ, 0U958ZZ, 0U9600Z, 0U960ZX, 0U960ZZ, 0U963ZZ, 0U9640Z, 0U964ZX, 0U964ZZ, 0U9670Z, 0U967ZX, 0U967ZZ, 0U9680Z, 0U968ZX, 0U968ZZ, 0UC50ZZ, 0UC53ZZ, 0UC54ZZ, 0UC57ZZ, 0UC58ZZ, 0UC60ZZ, 0UC63ZZ, 0UC64ZZ, 0UC67ZZ, 0UC68ZZ  Dataset-derived^a^ ICD-10-PCS: 0UC94ZZ, 0UQ40ZZ, 0W9G3ZZ, 0W9J0ZZ, 0W9N4ZZ, 10A00ZZ, 10A03ZZ, 10E0XZZ, 0D9W0ZZ, 0D9W4ZX, 0D9W4ZZ, 0DCW0ZZ, 0DCW4ZZ, 0DNW4ZZ, 0U504ZZ, 0U508ZZ, 0U514ZZ, 0U904ZZ, 0U910ZZ, 0UB00ZZ, 0UB04ZX, 0UB04ZZ, 0UB07ZZ, 0UB08ZX, 0UB10ZX, 0UB10ZZ, 0UB14ZZ, 0UB24ZZ, 0UB40ZZ, 0UB90ZX, 0UB90ZZ, 0UB94ZZ, 0UB98ZZ, 0UC04ZZ, 0UC14ZZ, 0UC90ZZ, 0UCF4ZZ, 0UJ30ZZ, 0UJ34ZZ, 0UJ80ZZ, 0UJ84ZZ, 0UJD0ZZ, 0UJD4ZZ, 0UL60ZZ, 0UL70ZZ, 0UQ00ZZ, 0UQ10ZZ, 0UQ90ZZ, 0UQ94ZZ, 0UT00ZZ, 0UT04ZZ, 0UT07ZZ, 0UT08ZZ, 0UT10ZZ, 0UT70ZZ, 0UT74ZZ, 0UT78ZZ, 0UT7FZZ, 0UT90ZL, 0UT90ZZ, 0UT94ZZ, 0UT97ZZ, 0UT98ZZ, 0UT9FZZ, 0W3J0ZZ, 0W3J4ZZ, 0W3R0ZZ, 0W3R4ZZ, 0W9G0ZZ, 0W9G4ZZ, 0W9J40Z, 0W9J4ZZ, 0WCG0ZZ, 0WCG4ZZ, 0WCH0ZZ, 0WCJ0ZZ, 0WCJ4ZZ, 0WJG0ZZ, 0WJG4ZZ, 0WJJ0ZZ, 0WJJ4ZZ, 0WJR4ZZ, 10D00Z0, 10D00Z1, 10D00Z2, 10J20ZZ, 10J23ZZ, 10J24ZZ, 10J27ZZ, 0UB50ZX, 0UB54ZX, 0UB57ZX, 0UB58ZX, 0UB60ZX, 0UB64ZX, 0UB68ZX, 0UB70ZX, 0UB70ZZ, 0UB74ZZ, 0UQ50ZZ, 0UQ60ZZ |
| Cesarean scar pregnancy | ICD-10-CM: O3421 |
| Exposure |  |
| Disability (by type) |  |
| Physical | ICD-10-CM: Q675, Q66, Q676, Q677, Q678, E343, E230, Q019, Q02, Q03, Q04, Q06, Q078, Q079, G901, Q75, Q76, Q77, Q78, Q79, Q72, Q73, Q74, Q71, Q05, Q70, E220, M45, M46, M863, M864, M865, M866, M500, M502, M503, M508, M509, M510, M512, M513, M514, M518, M519, M224, M232, M233, M234, M235, M238, M239, M15, M16, M17, M18, M19, M42, M91, M92, M93, M87, M80, M353, M05, M06, M47, G80, G90, G40, G81, G60, G11, G328, G57, G58, G35, G71, G72, G70, G54, G55, G36, G37, G95, G10, G23, G241, G242, G243, G244, G245, G248, G249, G25, G82, G83, G61, G62, G63, G318, G20, G21, I69, B91, G12, S020, S021, S023, S028, S029, S061, S062, S063, S064, S065, S066, S068, S069, S07, S77, S87, S970, Z993, Z998, S324, S325, S328, S140, S141, S240, S241, S340, S341, S343, M218, S78, S88, S980, S983, Z894, Z895, Z896, S48, S58, S684, Z891, Z892 |
| Sensory | ICD-10-CM: H90, H913, H918, H919, Q160, Q161, Q163, Q164, Q165, Q169, H54, H25, H26, H30, H31, Q111, Q112, Q131, Q133, Q138, Q150, H44, H201, H476, H40, H42, H55, E1031, E1032, E1033, E1035, E1131, E1132, E1133, E1134, E1135, H34, H35, H36 |
| Intellectual | ICD-10-CM: F840, F843, F845, F848, F849, Q860, F70, F71, F72, F73, F78, F79, Q90, Q91, Q920, Q921, Q922, Q925, Q927, Q928, Q929, Q93, Q971, Q992, Q998, Q851, Q861, Q871, Q8723, Q8731, Q878 |
| Outcomes |  |
| Route of surgery |  |
| Minimally invasive | ICD-10-PCS: 3, 4, 7, 8, or F in the 5^th^ digit |
| Open/Abdominal | ICD-10-PCS: 0 in the 5^th^ digit |
| Nature of surgery |  |
| Salpingostomy (tubal sparing) | ICD-10-PCS: 10D20ZZ, 10D24ZZ, 10D27ZZ, 10D28ZZ, 10T20ZZ, 10T23ZZ, 10T24ZZ, 10T27ZZ, 10T28ZZ, 0U9500Z, 0U950ZX, 0U950ZZ, 0U950ZZ, 0U953ZZ, 0U9540Z, 0U954ZX, 0U954ZZ, 0U9570Z, 0U957ZX, 0U957ZZ, 0U957ZZ, 0U9580Z, 0U958ZX, 0U958ZZ, 0U958ZZ, 0U9600Z, 0U960ZX, 0U960ZZ, 0U963ZZ, 0U9640Z, 0U964ZX, 0U964ZZ, 0U9670Z, 0U967ZX, 0U967ZZ, 0U9680Z, 0U968ZX, 0U968ZZ, 0UC50ZZ, 0UC53ZZ, 0UC54ZZ, 0UC57ZZ, 0UC58ZZ, 0UC60ZZ, 0UC63ZZ, 0UC64ZZ, 0UC67ZZ, 0UC68ZZ, 0UQ50ZZ, 0UQ60ZZ |
| Salpingectomy (tubal removal) | ICD-10-PCS: 0U550ZZ, 0U553ZZ, 0U554ZZ, 0U557ZZ, 0U558ZZ, 0U560ZZ, 0U563ZZ, 0U564ZZ, 0U567ZZ, 0U568ZZ, 0UB50ZZ, 0UB53ZZ, 0UB54ZZ, 0UB57ZZ, 0UB58ZZ, 0UB60ZZ, 0UB63ZZ, 0UB64ZZ, 0UB67ZZ, 0UB68ZZ, 0UT50ZZ, 0UT54ZZ, 0UT57ZZ, 0UT58ZZ, 0UT5FZZ, 0UT60ZZ, 0UT64ZZ, 0UT67ZZ, 0UT68ZZ, 0UT6FZZ, 0UB50ZX, 0UB54ZX, 0UB57ZX, 0UB58ZX, 0UB60ZX, 0UB64ZX, 0UB68ZX, 0UB70ZX, 0UB70ZZ, 0UB74ZZ |
| Hysterectomy | ICD-10-PCS: 0UB9, 0UT9 |
| Oophorectomy | ICD-10-PCS: 0UB0, 0UB1, 0UB2, 0UT0, 0UT1, 0UT2 |
| Blood transfusion | ICD-10-PCS: 30230, 30233, 30240, 30243  With H, K, L, M, N, P, R, T in the 6^th^ digit and 0 or 1 in the 7^th^ digit |
| Bilateral salpingectomy | ICD-10-PCS: 0UB7, 0UT7 |
| Tubal ligation | ICD-10-PCS: 0U57, 0UL7, 0UF7 |
| Baseline characteristics |  |
| Smoking status |  |
| Current smoker | ICD-10-CM: F17.2, Z72.0, O99.33 |
| Former smoker | ICD-10-CM: Z87.891 |
| Diabetes | ICD-10-CM: E10.0, E10.1, E10.9, E11.0, E11.1, E11.9, E12.0, E12.1, E12.9, E13.0, E13.1, E13.9, E14.0, E14.1, E14.9, E10.2–E10.8, E11.2–E11.8, E12.2–E12.8, E13.2–E13.8, E14.2–E14.8 |
| Hypertension | ICD-10-CM: I10, I11-I13, I15 |
| Cardiovascular disease | Binary composite indicator representing ≥1 of the following conditions |
| Congestive heart failure | ICD-10-CM: I09.9, I11.0, I13.0, I13.2, I25.5, I42.0, I42.5–I42.9, I43, I50, P29.0 |
| Cardiac arrhythmias | ICD-10-CM: I44.1–I44.3, I45.6, I45.9, I47–I49, R00.0, R00.1, R00.8, T82.1, Z45.0, Z95.0 |
| Valvular disease | ICD-10-CM: A52.0, I05–I08, I09.1, I09.8, I34–I39, Q23.0–Q23.3, Z95.2–Z95.4 |
| Pulmonary circulation disorders | ICD-10-CM: I26, I27, I28.0, I28.8, I28.9 |
| Peripheral vascular disorders | ICD-10-CM: I70, I71, I73.1, I73.8, I73.9, I77.1, I79.0, I79.2, K55.1, K55.8, K55.9, Z95.8, Z95.9 |
| Psychiatric illness | Binary composite indicator representing ≥1 of the following conditions |
| Depression | ICD-10-CM: F20.4, F31.3–F31.5, F32, F33, F34.1, F41.2, F43.2 |
| Alcohol use disorder | ICD-10-CM: F10, E52, G62.1, I42.6, K29.2, K70.0, K70.3, K70.9, T51, Z50.2, Z71.4, Z72.1 |
| Drug use disorder | ICD-10-CM: F11–F16, F18, F19, Z71.5, Z72.2 |
| Anxiety | ICD-10-CM F40, F41 |
| Obesity | ICD-10-CM E66 |
| Gynecologic disease | Binary composite indicator representing ≥1 of the following conditions |
| Endometriosis | ICD-10-CM N80 |
| Uterine fibroids | ICD-10-CM D25 |
| Abnormal uterine bleeding | ICD-10-CM N84.0, N84.1, N92, N93, N95.0 |
| Pelvic inflammatory disease | ICD-10-CM N70, N71, N73, N74 |
| Adnexal pathology | ICD-10-CM N83, D27, C56, C57 |

^a^ Hospitalization records with an ectopic pregnancy diagnosis code but without a prespecified surgical management code were manually reviewed by an obstetrician-gynecologist with expertise in administrative data coding to identify additional procedure codes related to atypical surgical management of ectopic pregnancy for cohort inclusion.

**eTable 2. Characteristics of National Inpatient Sample records for patients with and without disabilities who had an obstetric delivery between 2016-2021**

| Characteristic | No Disability  No. (%) | Disability  No. (%) | ASD |
| --- | --- | --- | --- |
| Total | N = 4,255,022 | N = 62,486 (1.4) | -- |
| Age |  |  |  |
| 15-19 years | 205,514 (4.8) | 2,476 (4.0) | 0.04 |
| 20-24 years | 815,421 (19.2) | 10,966 (17.5) | 0.04 |
| 25-29 years | 1,225,280 (28.8) | 16,723 (26.8) | 0.05 |
| 30-34 years | 1,237,094 (29.1) | 18,261 (29.2) | 0.00 |
| 35-39 years | 638,457 (15.0) | 11,267 (18.0) | 0.08 |
| 40-44 years | 133,256 (3.1) | 2,793 (4.5) | 0.07 |
| Race/ethnicity |  |  |  |
| Asian/Other | 467,316 (11.0) | 4,541 (7.3) | 0.13 |
| Black | 612,082 (14.4) | 9,380 (15.0) | 0.02 |
| Hispanic | 862,506 (20.3) | 9,186 (14.7) | 0.15 |
| White | 2,142,863 (50.4) | 37,294 (59.7) | 0.19 |
| Missing | 170,255 (4.0) | 2,085 (3.3) | 0.04 |
| Insurance Payer |  |  |  |
| Medicaid or Medicare | 1,817,255 (42.7) | 30,003 (48.0) | 0.11 |
| Private Insurance | 2,205,900 (51.8) | 29,957 (47.9) | 0.08 |
| Other | 226,745 (5.3) | 2,469 (4.0) | 0.07 |
| Missing | 5,122 (0.1) | 57 (0.1) | 0.01 |
| Median household income for ZIP code |  |  |  |
| Quartile 1 (low) | 1,174,434 (27.6) | 17,724 (28.4) | 0.02 |
| Quartile 2 | 1,064,041 (25.0) | 15,783 (25.3) | 0.01 |
| Quartile 3 | 1,043,543 (24.5) | 15,203 (24.3) | 0.00 |
| Quartile 4 (high) | 935,969 (22.0) | 13,210 (21.1) | 0.02 |
| Missing | 37,035 (0.9) | 566 (0.9) | 0.00 |
| Comorbidities |  |  |  |
| Diabetes | 62,886 (1.5) | 2,724 (4.4) | 0.17 |
| Hypertension | 9,265 (0.2) | 448 (0.7) | 0.07 |
| Cardiovascular disease | 80,468 (1.9) | 2,781 (4.5) | 0.15 |
| Depression | 259,251 (6.1) | 8,568 (13.7) | 0.26 |
| Anxiety | 216,268 (5.1) | 9,093 (14.6) | 0.32 |
| Substance Use Disorder (Alcohol or Drugs) | 172,493 (4.1) | 4,890 (7.8) | 0.16 |
| Obesity | 600,647 (14.1) | 11,841 (18.9) | 0.13 |
| Endometriosis | 6,218 (0.1) | 203 (0.3) | 0.04 |
| Uterine fibroids | 63,650 (1.5) | 1,269 (2.0) | 0.04 |
| Abnormal uterine bleeding | 3,030 (0.1) | 66 (0.1) | 0.01 |
| Pelvic inflammatory disease | 31,103 (0.7) | 561 (0.9) | 0.02 |
| Adnexal pathology | 27,106 (0.6) | 553 (0.9) | 0.03 |
| Smoking status |  |  |  |
| Never | 3,792,029 (89.1) | 49,760 (79.6) | 0.26 |
| Former smoker | 248,529 (5.8) | 6,319 (10.1) | 0.16 |
| Current smoker | 214,464 (5.0) | 6,407 (10.3) | 0.20 |

ASD: Absolute standardized difference, > 0.10 indicates an important difference between women with versus without disability. No. (%): unweighted number (weighted proportion).

**eTable 3. Outcomes of surgically managed ectopic pregnancy by disability status, National Inpatient Sample 2016-2021, further assessed by additionally adjusting for comorbidities and excluding cases of Cesarean scar pregnancy**

|  | Adjusted RR (95% CI) | | | | | |
| --- | --- | --- | --- | --- | --- | --- |
| Disability | Main Analyses | | Adjusted for  Comorbidities | | Excluding Cases of Cesarean Scar Pregnancy | |
| Open (vs. Minimally Invasive) Route of Surgery | | | | | | |
| No Disability | 1.00 | (Reference) | 1.00 | (Reference) | 1.00 | (Reference) |
| Any Disability | 0.98 | (0.83, 1.17) | 0.98 | (0.82, 1.17) | 0.99 | (0.83, 1.18) |
| Tubal Removal (vs. Tubal Sparing) Procedure | | | | | | |
| No Disability | 1.00 | (Reference) | 1.00 | (Reference) | 1.00 | (Reference) |
| Any Disability | 0.96 | (0.89, 1.04) | 0.96 | (0.89, 1.04) | 0.96 | (0.89, 1.04) |
| Prolonged LOS |  |  |  |  |  |  |
| No Disability | 1.00 | (Reference) | 1.00 | (Reference) | 1.00 | (Reference) |
| Any Disability | 1.34 | (1.03, 1.74) | 1.22 | (0.93, 1.58) | 1.34 | (1.02, 1.74) |
| Blood Transfusion |  |  |  |  |  |  |
| No Disability | 1.00 | (Reference) | 1.00 | (Reference) | 1.00 | (Reference) |
| Any Disability | 0.95 | (0.66, 1.37) | 0.91 | (0.63, 1.33) | 0.95 | (0.66, 1.37) |
| Composite extensive procedures^a^ | | |  |  |  |  |
| No Disability | 1.00 | (Reference) | 1.00 | (Reference) | 1.00 | (Reference) |
| Any Disability | 1.49 | (1.11, 2.00) | 1.28 | (0.96, 1.70) | 1.50 | (1.11, 2.02) |
| Hysterectomy | | | | | | |
| No Disability | 1.00 | (Reference) | 1.00 | (Reference) | 1.00 | (Reference) |
| Any Disability | 1.75 | (0.91, 3.36) | 1.51 | (0.80, 2.84) | 1.78 | (0.89, 3.59) |
| Oophorectomy | | | | | | |
| No Disability | 1.00 | (Reference) | 1.00 | (Reference) | 1.00 | (Reference) |
| Any Disability | 1.43 | (0.96, 2.13) | 1.21 | (0.82, 1.80) | 1.43 | (0.96, 2.14) |
| Bilateral Salpingectomy | | | | | | |
| No Disability | 1.00 | (Reference) | 1.00 | (Reference) | 1.00 | (Reference) |
| Any Disability | 1.30 | (0.71, 2.37) | 1.18 | (0.65, 2.15) | 1.25 | (0.66, 2.36) |

CI: confidence interval. RR: risk ratio. All models controlled for patient age, race/ethnicity, insurance type, median household income quartile, smoking status, and year of surgery. Comorbidities include cardiometabolic diseases, psychiatric illness, obesity, and gynecologic diseases.

^a^ Use of more extensive procedures than standard, including hysterectomy, oophorectomy (bilateral or unilateral), bilateral salpingectomy, or tubal ligation.
